# Genomic evidence of recent hybridization between sea turtles at Abrolhos Archipelago and its association to low reproductive output

**DOI:** 10.1038/s41598-020-69613-8

**Published:** 2020-07-30

**Authors:** Larissa Souza Arantes, Lucas Cabral Lage Ferreira, Maximilian Driller, Fernando Pedro Marinho Repinaldo Filho, Camila Junqueira Mazzoni, Fabrício Rodrigues Santos

**Affiliations:** 1 Departamento de Genética, Ecologia e Evolução, Instituto de Ciências Biológicas, Universidade Federal de Minas Gerais (UFMG), Avenida Antônio Carlos, 6627, Belo Horizonte, MG 31270-010 Brazil; 2 Parque Nacional Marinho dos Abrolhos, ICMBio, Ministério do Meio Ambiente, Brasilia, Brazil; 3 Berlin Center for Genomics in Biodiversity Research (BeGenDiv), Königin-Luise-Straße 6-8, 14195 Berlin, Germany; 4 Evolutionary Genetics Department, Leibniz Institute for Zoo and Wildlife Research (IZW), Alfred-Kowalke-Straße 17, 10315 Berlin, Germany

**Keywords:** Genetics, Genetic hybridization

## Abstract

Hybridization between sea turtle species occurs with particularly high frequency at two adjacent nesting areas in northeastern Brazil. To understand the outcomes of hybridization and their consequences for sea turtle conservation, we need to evaluate the extent of hybridization occurrence and possible deleterious effects in the hybrid progeny. Thus, we investigated the hypothesis of the existence of a new hybrid spot offshore of Brazil’s northeastern coast. The Abrolhos Archipelago is surrounded by the largest and richest coral reefs in the South Atlantic and is known to be a nesting site for loggerhead turtles (*Caretta* *caretta*). In this study, we performed a multidisciplinary investigation into levels of hybridization in sea turtles and their reproductive output in the Abrolhos beaches. Genetic data from mitochondrial DNA (mtDNA) and six autosomal markers showed that there are first-generation hybrid females nesting in Abrolhos, resulting from crossings between hawksbill males (*Eretmochelys* *imbricata*) and loggerhead females, and backcrossed hatchlings from both parental species. The type and extent of hybridization were characterized using genomic data obtained with the 3RAD method, which confirmed backcrossing between F1 hybrids and loggerhead turtles. The reproductive output data of Abrolhos nests suggests a disadvantage of hybrids when compared to loggerheads. For the first time, we have shown the association between hybridization and low reproductive success, which may represent a threat to sea turtle conservation.

## Introduction

Hybridization may play an important role in the evolutionary history of species^1^. However, predicting the outcomes of crossings between genetically differentiated species or populations is extremely hard, as it depends on various factors that shape each particular case, such as the divergence between the species involved in hybridization, the extent of the process and the fitness impact on hybrids, which is determined by complex genetic mechanisms^2^. A reduction in hybrid fitness—also known as outbreeding depression—happens when hybridization leads to lower reproductive success in hybrids relative to the parental species. When significant outbreeding depression is concurrent with continual hybridization events, this can lead to a decrease in the population growth rate of the rarer species to a level below that required for replacement, a phenomenon known as demographic swamping^3^. In extreme cases, the complete loss of reproductive potential for one of the species may occur, leading to localized extinction events^4^, which have the potential to impact neighboring populations in a cascading fashion. Alternatively, when hybrid fitness decrease is not significant, multiple generations of hybrids can occur, resulting in the loss of genetically pure individuals for one of the species while preserving the genetic material from that same species in introgressed individuals, which is known as genetic swamping^2^.

Although hybridization is generally considered a natural phenomenon happening in about 25% of plants and 10% of animals^1^, it becomes a matter of concern when it is a consequence of anthropogenic actions and/or involves endangered species^4,5^. Human-mediated environmental change, including habitat modification and exotic species introductions, may favor interspecific crossings and lead to demographic or genetic swamping, threatening species integrity, especially in small populations or rare species^6^. Considering the large-scale declines in many animal populations, resulting in the increasing scarcity of conspecific mates, the early recognition of hybrids should be a priority in the conservation management of small populations^7^.

According to the World Conservation Union (IUCN) Red List of Threatened Species, six out of the seven species of sea turtles are considered globally as either endangered, critically endangered or vulnerable. Sea turtles comprise a group with deep divergence times, reaching up to 110 million years^8^. Occasional hybridization has been detected between very divergent species, such as between Carettini and Chelonini turtles, which have 63 million years of separation^9^. However, hybridization has been shown to occur more frequently in two northeastern spots along the Brazilian coast between sea turtle species with separation times of up to 29 million years, involving mainly three Carettini species: hawksbill (*Eretmochelys* *imbricata*), loggerhead (*Caretta* *caretta*) and olive ridley (*Lepidochelys* *olivacea*)^10^. In both areas, loggerhead turtles—which have an increasing population trend in the South Atlantic—hybridize with either hawksbill (in northern Bahia state)^11^ or olive ridley turtles (in Sergipe state)^12^.

Hybrid females found nesting in both Brazilian populations are first generation (F1) hybrids, which suggests that the hybridization process is recent and probably associated with environmental changes and global population declines in sea turtles^13^. Backcrossed hatchlings have also been reported, confirming that F1 hybrids are fertile and introgression may be possible^10,13^. Despite the confirmed viability of hybrid F1 females, it has been shown that their nests in northern Bahia have a lower success rate of hatching emergence, whilst other reproductive output parameters, such as hatchling production per clutch, clutch frequency and breeding frequency remained similar to the parental species^14^. Furthermore, no negative effect of hybridization was found on hatchling viability^15^. Nonetheless, little is known about sea turtle hybrids in other life stages in terms of survivorship, migratory behavior, feeding preferences, reproductive fitness and mate choice, such that the temporal and spatial extent of hybridization consequences for sea turtle populations remains unclear^13^.

Abrolhos Archipelago, which supports the largest and richest coral reefs in the South Atlantic and the world's largest rhodolith beds^16,17^, is located 70 km off shore from Bahia state and 600 km south of Praia do Forte, where loggerhead and hawksbill hybrids reach up to 42% of the identified hawksbill nesting population^11^. Besides being an important feeding area for hawksbill and green turtles (*Chelonia* *mydas*), respectively attracted by the richness of invertebrates and seagrasses^18–20^, Abrolhos is also a nesting area for loggerhead turtles, which lay their eggs on the short sandy beaches of Redonda and Santa Bárbara islands^21^. Recent nesting monitoring on the islands has identified a mean of 34 nests per reproductive season between the years of 2015 and 2018^20,22^. In 2015, hawksbill turtles were also reported to be nesting in the archipelago, but the mixed morphology of the nesting females suggested that they could in fact be hybrids^22^. However, no genetic analysis has ever been carried out to test the hypothesis of the existence of hybrids in Abrolhos. Furthermore, no hybrids were detected in the only population genetics study of hawksbill juveniles (N = 65) hand-captured by divers in the Abrolhos Archipelago^23^.

The proportion of eggs that produced live hatchlings, referred to as hatching success (HS), ranged from 25.4% in 2015/2016^22^ to 46.8% in 2016/2017 in Abrolhos^20^. The annual average HS of loggerhead turtles in Espírito Santo, Bahia and Rio de Janeiro states in Brazil was 79.9%^24^^,^ 73.1%^25^ and 76.74%^26^^,^ respectively, which demonstrated that the HS in Abrolhos is relatively low. This could be related to the features of Abrolhos’ beaches, which are very short, covered by boulders and grasses, frequently disrupted by tides, and composed of a mixture of carbonate debris and rock fragments^27^. The difficulty finding appropriate nesting sites in Abrolhos is confirmed by the low success rate of nesting attempts, with 40% of sea turtles emerging from the ocean onto the beach during the reproductive season of 2017/2018 returning to the ocean without nesting^20^. Alternatively, other biotic factors associated with the sea turtle population of Abrolhos may also affect the HS, such as the still unverified presence of interspecific hybridization^28^.

In this paper, we tested the hypothesis of the existence of sea turtle hybrids in the Abrolhos Archipelago through different levels of genetic analysis. We used a combination of mtDNA and six species-diagnostic autosomal loci to identify hybrids, backcrosses and pure parental species. To confirm the power of our molecular assay in establishing the generation and type of crosses of later hybrids, we used genomic data obtained through the 3RAD (Restriction site Associated DNA) method. We also assessed the reproductive output of the Abrolhos population and tested whether interspecific hybridization is associated with low hatching success in Abrolhos. These analyses provide new insights into the conservation of sea turtle species, particularly the Abrolhos population.

## Methods

### Study area

**Figure 1:**
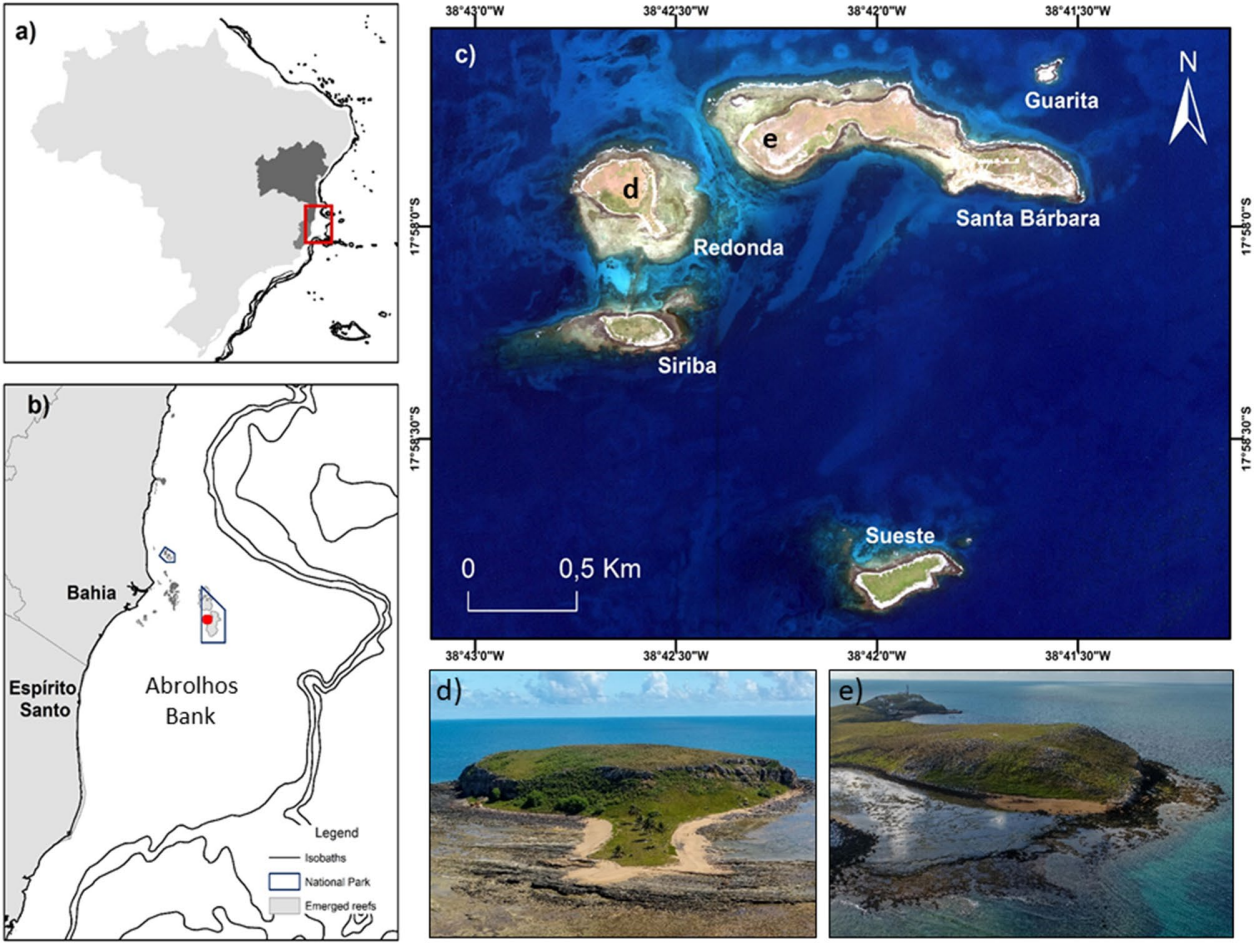
The Abrolhos Archipelago. It is located along the Brazilian coast (**a**), in the Abrolhos bank in south Bahia state (**b**) and encompasses five small islands (**c**). The nesting sites of sea turtles include short sand stretches in Redonda (**d**) and Santa Bárbara (**e**) islands.

This study was performed in the Abrolhos Archipelago (17° 20′–18° 10′ S and 38° 35′–39° 20′ W), a set of five small volcanic islands situated on the Abrolhos Bank, 70 km offshore from Bahia state (Fig. 1). The Abrolhos Bank is an expansion of the southern part of the Eastern Brazilian continental shelf that comprises mangroves, seagrass and algae bottoms, euphotic and mesophotic coral reefs with high levels of endemism and unique mushroom-shaped coralline pinnacles^29,30^. The archipelago is part of the Abrolhos National Marine Park, which is responsible for the conservation of its unique ecosystem.

The nesting area of sea turtles includes short sand stretches on the islands of Santa Bárbara and Redonda. Santa Bárbara is the largest island in the archipelago and contains two gravelly and sandy beaches on its northern and southern sides. Beaches on Redonda Island are strongly tide-influenced, comprised of longer stretches of coral sand, with beach width ranging between 0 and 5 m. Fringing reefs surround most of the islands^16^. The reproductive season of sea turtles in the Abrolhos Archipelago occurs between September and March, a period characterized by hot temperatures consistent with the tropical, humid climate of Abrolhos.

### Sampling and data collection

Sampling and data collection was performed by the Abrolhos National Marine Park team applying the methodology established by Projeto TAMAR^25,31^. Sporadic patrols were conducted nightly along the beaches of the Santa Bárbara and Redonda islands between September and March from 2015 to 2019 (i.e. four seasons, including 2015/2016, 2016/2017, 2017/2018 and 2018/2019). Nesting females encountered were double tagged with Inconel metal tags on the front flippers and their curved carapace length (CCL) and curved carapace width (CCW) measured^31^. During the 2015/2016 reproductive season, the females (N = 6) found during oviposition were sampled through a small piece of skin taken from either the anterior flipper or the neck region using a sterilized scalpel. These samples were stored in EtOH 70% at − 20 °C and deposited in the Taxonomic Collections Center of the Federal University of Minas Gerais (UFMG), Belo Horizonte, Brazil.

Patrols during the morning were conducted daily on the beaches of Santa Bárbara and every 2 days in Redonda. Based on the tracks and nesting signs, nests were identified by carefully probing with a stick and then digging by hand to locate the eggs. All nests were marked with a unique number and monitored until hatchling emergence, indicative of a successful nesting event. Once hatchling tracks emerging from the nest were detected, the hatched nests were excavated, the species of hatchlings were morphologically identified whenever possible, and the number of dead hatchlings and unhatched eggs was quantified. The hatched eggshells were counted to estimate the number of live hatchlings produced in each nest. The date of each nesting event was recorded to verify the temporal nesting distribution in the Abrolhos Archipelago. During the 2017/2018 reproductive season, samples were taken from between one and three dead hatchlings from each of 20 distinct nests by collecting anterior flipper fragments as described above.

Nests were divided into two groups based on the classification of the nesting female as either loggerhead or hybrid. When a female was encountered while nesting, the nest classification was done morphologically, based on the size of the female’s head, shape of the beak, number of lateral scutes and shape of the shell. The loggerhead turtle has a heart-shaped carapace, a rather long and very broad head and five pairs of lateral scutes with the anterior pair touching the pre-central scute, as described by Conceição et al.^32^. The hawksbill turtle has a medium-sized head, strong horny beak, depressed oval carapace, shell-imbricated scutes, and four pairs of lateral scutes on the carapace with the anterior pair not touching the pre-central scute. First generation hybrids usually exhibit a different pattern of scutes on the shell, and have a medium-sized head, pointy beak and depressed oval carapace, similar to hawksbill turtles (see Supplementary Figure S1).

Unlike for females, the possibility to perform a morphological species assignment on hatchlings depends on the decomposition state of the dead individuals, as well as the number of and variation among individuals found in the nest. Therefore, whenever a nest was found without a nesting female encounter, nest classification was done based on the genetic species assignment of hatchlings by analyzing between one (when only multilocus analysis was performed—see “Genetic analysis” section) and three (when 3RAD was also performed—see 3RAD-related sections) hatchlings per nest. We assumed that properly identifying one hatchling per nest as either pure loggerhead or a backcross was enough to determine if the mother was either a loggerhead turtle or a F1 hybrid. As all hatchlings and females analyzed in this work were collected during different seasons (2017/2018 and 2015/2016, respectively), no information is available connecting parentage and nests among the samples genetically analyzed.

The incubation period (IP) was calculated as the period between oviposition and hatchling emergence. The clutch size (CS) was determined by nest excavation after emergence, counting the total number of unhatched eggs, dead hatchlings and the potential number of live hatchlings (estimated as the difference between all found eggshells and dead individuals) per nest. The hatching success (HS) was calculated through the proportion of eggs that produced live hatchlings reaching the beach surface compared to the total number of laid eggs. The female body size was calculated using the first measurement of CCL and CCW recorded for each animal (N = 18). We compared these parameters between two different groups: (1) loggerhead turtles and (2) hybrids, including individuals for which species assignment was confirmed either by genetic analysis (N = 32) or based on the morphology of females encountered during nesting (N = 15). To test for differences between groups, statistical analysis was performed with nonparametric t test (Mann–Whitney U test) implemented in GraphPad Prism 5.0 software.

### Genetic analysis

We extracted genomic DNA from the tissue samples with either a modified phenol–chloroform protocol^33^ or the DNeasy Blood and Tissue kit (Qiagen). In order to determine the type of hybrid, we used a multilocus sequencing approach developed by Arantes et al.^13^^,^ in which 14 loci presenting species-specific diagnostic alleles/haplotypes (i.e. high-quality phased sequences) were selected from double-digest RADseq (ddRADseq) data produced the same way for both loggerhead and hawksbill turtles. Since the primers developed were based on data generated for both species, the reproducibility was very high. For the present work, we selected the six nuclear DNA (nDNA) loci (421; 856; 3,061; 64,188; 76,958; and 109,472) presenting the highest species assignment accuracy in the previous study^13^. Based on other cases of hybridization in Brazil, we expected to find hybrids of up to two generations (F1 and backcrossing). Considering the 50% chance of detecting homospecific or heterospecific loci in a backcrossed individual, we estimated an error rate of assignment of a hybrid individual with single nucleotide polymorphism (SNP) data from these six markers of 1.56%. Posterior genomic analysis confirmed the hybrid categorization of some individuals. We conducted polymerase chain reactions (PCRs) as described in Arantes et al.^13^. Amplicons were sequenced on an ABI 3130xl DNA sequencer (Applied Biosystems).

High-quality consensus sequences were used in the alignments performed with the ClustalW algorithm in the MEGA 7 software^34^. We included in the alignment for each marker all known loggerhead and hawksbill haplotypes previously determined by Arantes et al.^13^. Gametic phases for nDNA markers were resolved with the PHASE algorithm^35^ and the haplotypes inference was done with the DnaSP v5 program^36^.

Diagnostic sites (SNPs) were used to test the hybrid index (HI) in the R package gghybrid^37^^,^ which calculates the proportion of allele copies coming from parental reference sets using a Bayesian algorithm^38,39^. As references for parental species, we included five loggerhead and five hawksbill turtles from different populations along the Brazilian coast analyzed by Arantes et al.^13^. We ran HI estimations using 50,000 Markov Chain Monte Carlo (MCMC) iterations after a 10,000 MCMC burn-in period.

We also analyzed the control region of mtDNA using the primers LCM 15,382 and H950^40^. The PCR included 200 μM dNTP, 0.3 units (U) Platinum Taq DNA polymerase (Invitrogen by Life Technologies), 1.5 mM of MgCl_2_, 0.5 mg/mL of Bovine Serum Albumin (BSA), 0.5 μM forward and reverse primers and 15 ng genomic DNA in 1X reaction buffer in a final volume of 10 μl. PCR cycling conditions were performed with one initial denaturation cycle of 95 °C for 5 min, 35 cycles of denaturation at 95 °C for 30 s, annealing at 50 °C for 30 s, extension at 72 °C for 1 min, and a final extension at 72 °C for 7 min. mtDNA haplotypes were identified running a BLAST search as implemented in the NCBI database (https://www.ncbi.nlm.nih.gov). Haplotype identification was also checked against the database of The Archie Carr Center for Sea Turtle Research (https://accstr.ufl.edu/resources/mtdna-sequences) and haplotypes were named as assigned by Shamblin et al^41^.

### 3RAD library construction and sequencing

We used the 3RAD protocol as described by Bayona-Vásquez et al.^42^ to analyze hatchlings from six different nests collected at Abrolhos. Briefly, we digested 100 ng of genomic DNA with the enzymes MseI, EcoRI and CviQI (New England Biolabs) at 10 U/μL and the specific combination of adapters for each sample for 1 h at 37 °C. We proceeded with the ligation of adapters containing in-line barcodes on both P5 and P7 adapters^42^^,^ immediately after the digestion. Barcoded samples were equimolarly pooled and cleaned with 0.8X CleanPCR magnetic beads (GC biotech). Subsequently, we performed the size selection of fragments between 350 and 450 bp with Blue Pippin using a 1.5% cassette and the R2 marker (Sage Science). This range was chosen based on an in silico digestion analysis of the ~ 2.2 Gb green sea turtle draft genome currently available (GenBank accession number GCF_000344595.1). Using a dedicated python script, we have estimated ~ 21,300 loci based on the three enzymes and size range used to build our 3RAD libraries.

The libraries were submitted to a single-cycle PCR to add the Tru5-8N primer^43^^,^ followed by a ten-cycle indexing PCR, in which we used a P5 short primer and P7 indexing primer to complete the library construction. The final library was characterized with qPCR using the KAPA Library Quantification Kit (Kapa Biosystems) and checked with the Agilent 2100 Bioanalyzer High Sensitivity DNA. The fragments were sequenced using 150 bp paired end reads in one partial lane of the HiSeq 4000 platform (Illumina) with the TruSeq 300-cycle Kit, aiming at a minimum of 20 × coverage per individual sequenced.

### 3RAD processing and variant detection

Illumina reads were demultiplexed based on P7 indexes and the raw sequence quality was checked with Fastqc^44^ and Multiqc^45^. The software FLEXBAR^46^ was used to demultiplex the P5 in-line barcodes. To remove PCR replicates, we used the python script Filter_PCR_duplicates.py, which recognizes identical combinations of i5 sequence tags and the first 100 bp of each read pair sequence and keeps only a single representative copy of the pair in the output. We used the software PEAR^47^ to merge reads pairs with overlapping regions of at least 30 bp. All merged reads less than 240 bp were considered as short fragments (i.e. out of the size selection range) and were removed from the subsequent analysis. The unassembled reads were kept in the analysis as paired-end reads, filtered for quality (Q > 30) and trimmed to a maximum length of 130 bp using Trimmomatic^48^. The reverse read presented low quality in the first 6 bp, which were therefore removed from the sequences for subsequent analysis. This probably occurred due to low sequence diversity at the EcoRI recognition site, which was identical for virtually all reads. We used the Check_Restriction_Site.py script to select only read pairs digested by MseI, filtering out any fragment digested by CviQI. Finally, read pairs containing an internal complete restriction site of MseI, EcoRI or CviQI were removed using the Filter_Reads.py script. The number of reads filtered out in each step is shown in Supplementary Figure S2.

3RAD sequences were analyzed using the green turtle draft genome as a reference^49^. Reads were mapped using Bowtie2 software^50^ with default parameters and the flags "-no-mixed" and "-no-discordant" to ensure that only paired reads aligned to the same locus would be present in the SAM files. Mapped reads were then analyzed with the Stacks reference-based pipeline^51^. The gstacks.pl module was used to call variant sites within the population for each locus and genotype each individual at each identified SNP. The populations.pl module was used to filter population parameters, allowing the inclusion of a locus in the final data set only if the locus was genotyped in at least 40% of individuals within a population and was present in all 3 populations.

Since haplotypes increase the power to detect population structure regarding SNPs^52^^,^ we used the Create_Haplotype_Structure.py script to code the haplotypes as multi-allelic loci for the Bayesian clustering analysis performed in the software STRUCTURE^53^. The script uses Stacks output files as well as the alignments in SAM format to filter the set of loci, based on a given read minimum coverage (– readCOV), a minimum number of populations covering a locus (– popCOV), as well as a percentage of individuals within a population to cover a locus (– intraCOV). Additionally, reads with undefined positions were filtered out. We used the following parameters: readCOV = 6, popCOV = 3, and intraCOV = 0.7.

The haplotype data was used to test the assignment of individuals to populations assuming the admixture model in STRUCTURE. We included individuals of loggerhead (N = 5) and hawksbill (N = 5) turtles as a control for species-specific haplotypes and hybrid diagnostics. Using independent allele frequencies, ten independent runs for each K value (from K = 1 to K = 5) were performed with 500,000 MCMC repeats after a 100,000 burn-in period. We summarized the replicates and visualized the STRUCTURE-estimated membership coefficients using CLUMPAK^54^.

The parental species and the hybrid categories (F1, F2, backcrosses) of each hybrid individual were determined with the program NewHybrids v. 1.1 Beta3^55^. The analysis was done using a burn-in period of 10,000 followed by 50,000 MCMC iterations with Jeffrey option and no priors. We restricted the dataset to the first 300 loci, due to the NewHybrids’ limited processing capacity. We also tested NewHybrids with a different set of 300 loci that were selected randomly to ensure the consistency of the results. The R package HybridDetective was used to plot the NewHybrids analysis^56^.

## Results

### Hybrid identification

A total of 47 nests were analyzed in this study and classified into two groups: loggerhead turtles and hybrids. For 28 nests, 17 different females were encountered while nesting (average of 1.6 nests per female) and were therefore classified based on the female’s morphology. The remaining 19 nests, plus one nest with assigned mother, were tested genetically with a combination of mtDNA control region and autosomal multilocus analyses. Six of the 19 remaining nests were also analyzed using 3RAD genomic data. A representation of all nests analyzed in this study and the types of species and hybrid identification performed is shown in Supplementary Figure S3.

Eleven females (found in 20 nests) were classified as loggerhead turtles and six females (found in eight nests) as hybrids. Six females were also tested molecularly with the multilocus approach and in all cases the morphological classification was confirmed (five loggerhead turtles and one hybrid). For the 19 nests with no assigned mother, female classification was performed based on hatchling genetic analysis (between one and three tested per nest). Eleven nests were classified as loggerheads and eight nests as hybrids. The classification of the mothers as hybrids was based on the identification of hatchlings as backcrosses with either loggerhead turtles or hawksbill turtles (see below for more details). Nests were assumed to be properly represented by one hatchling because our main goal was to distinguish nests from hybrid versus pure loggerhead females. Once a purely loggerhead hatchling is found, the mother can be assumed to be pure and once a second generation hybrid is found, the mother can be assumed to be a hybrid. First generation hybrids would also indicate a pure parental species mother, but these were not found among the hatchlings (see below for details of each genetic analysis).

The analysis of the mtDNA control region indicated that all analyzed sea turtles from Abrolhos (Supplementary Table S1) presented the mtDNA haplotype CC-A4.1 (GenBank accession number EU179457.1), which is typically found in loggerhead turtles of rookeries in the states of Sergipe, Bahia, Espírito Santo and Rio de Janeiro^41^. It is therefore not possible to distinguish the Abrolhos population from other Brazilian rookeries based on the mtDNA control region.

**Figure 2:**
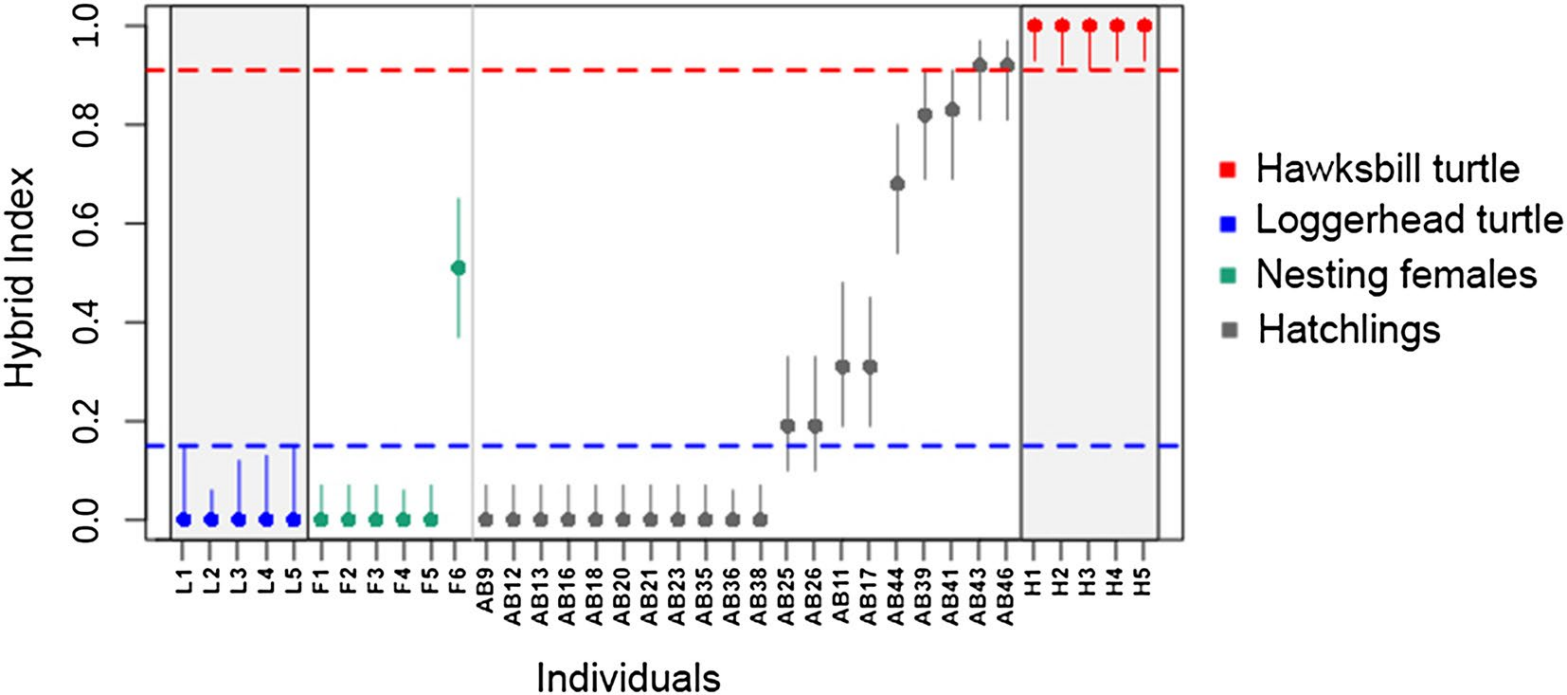
Bayesian estimates of the hybrid index (HI) for each individual analyzed in Abrolhos Archipelago. Blue and red points represent the parental reference sets of loggerhead and hawksbill turtles, respectively. Cyan points represent nesting females and grey points are hatchlings from 20 nests from Abrolhos. The HI was estimated with gghybrid. HI estimated values equal to 0.0 denote pure loggerhead turtles and 1.0 denote pure hawksbill individuals; lines represent 95% confidence intervals.

We also analyzed the sequences of a set of six nuclear markers (Supplementary Table S1) that have consistently shown divergent haplotypes between loggerhead and hawksbill turtles, but for which the amplification of hybrid loci is still highly efficient^13^. Bi-allelic SNP data analyzed through Bayesian hybrid-index confirmed the morphological assignment of all six females (Fig. 2). The only hybrid female presented one hawksbill turtle allele and one loggerhead turtle allele for all six nuclear markers, as well as the loggerhead mtDNA haplotype CC-A4.1. Thus, the results suggest that this individual is a first generation hybrid between a hawksbill male and a loggerhead female (HxL).

Of the 20 hatchlings analyzed with the multilocus approach, one from each nest, the hybrid index showed that 11 hatchlings are loggerhead turtles, four hatchlings are backcrosses of HxL with loggerhead turtles and five hatchlings are backcrosses of HxL with hawksbill turtles (Fig. 2).

Hatchling morphological classification was carried out at all nests for which morphological evidence was available (16 of the 20 nests). The genetic analyses confirmed the morphology of only 12 individuals (five loggerheads and seven putative hybrids), while four hatchlings morphologically identified as putative hybrids were in fact genetically classified as pure loggerhead turtles.

3RAD data was generated for ten control individuals (five loggerhead and five hawksbill turtles) as well as for 14 hatchlings from six different nests (see Supplementary Table S1 for a complete list of individuals tested). Of the ten control individuals, three included foraging hawksbill juveniles from Abrolhos. The sequencing of the 24 individual 3RAD libraries yielded an average of 830,000 reads per individual (plus or minus one standard deviation: SD ± 460 k) after quality filtering steps (see Methods for more details on the filters). The average per-sample locus coverage was 39.8x ± 22.2x. The datasets obtained with Stacks initially generated 23,406 loci and retained 4,534 after the application of all filters. A total of 13,882 variant sites were combined in 2,405 loci, which were used in the analyses. The average number of haplotypes per locus was 3.55 ± 1.3 and the average number of variable sites per locus was 3.99 ± 2.2. It is important to note that these numbers refer to the analysis of both species together. The proportion of missing data per individual for loggerhead and hawksbill turtles used as controls was on average 5.45% and 10.3% respectively, while across all fourteen hatchlings, the average missing data per individual was 14.9%.

**Figure 3:**
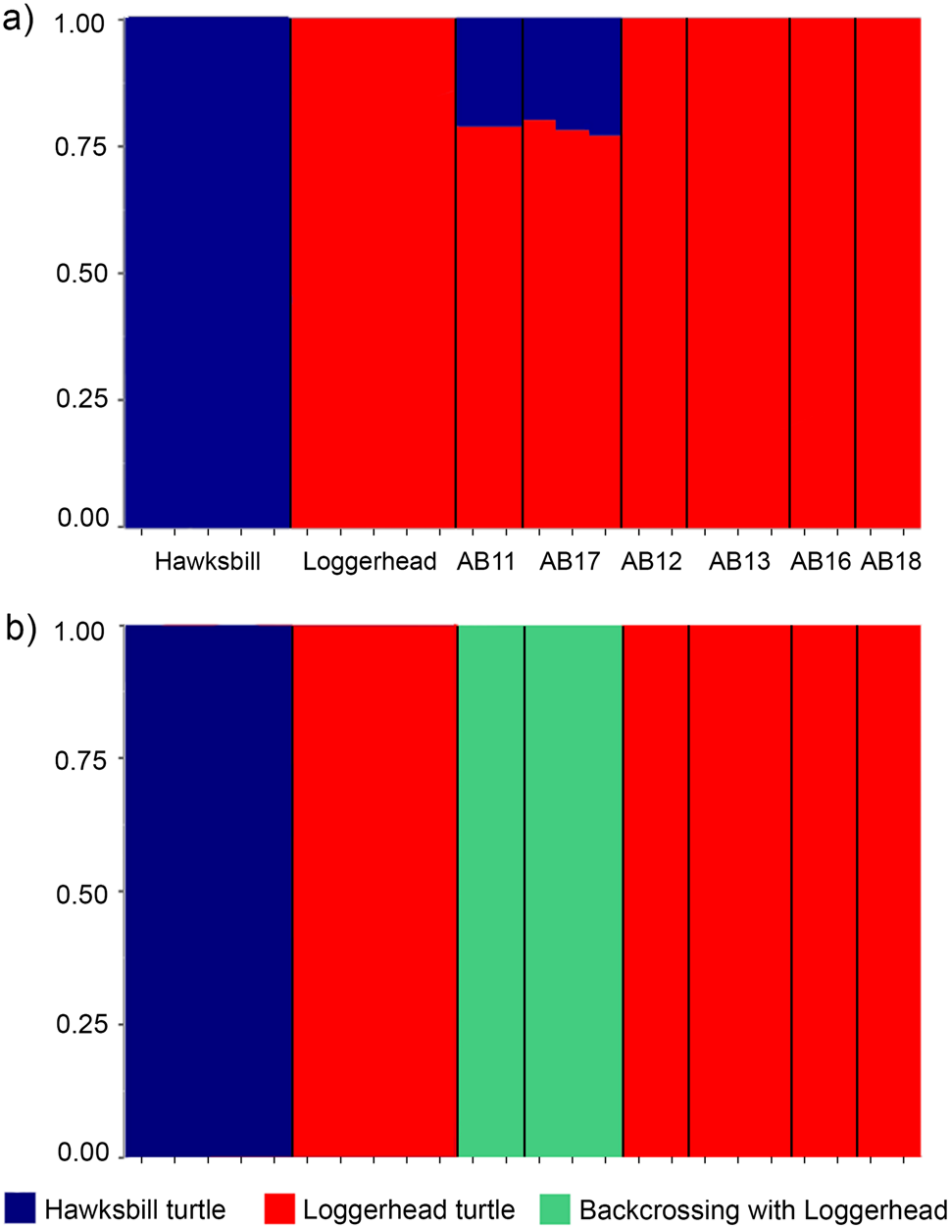
Genetic structure of parental species and their hybrids based on 3RAD data. Admixture proportions based on two clusters (K = 2) estimated by STRUCTURE (**a**) and assignment of hybrid class by NewHybrids (**b**). The plots include the five hawksbill and five loggerhead turtles and 14 hatchlings from six different nests from Abrolhos. The order of the individuals within the different groups is the same in both plots.

The genomic data analysis using the STRUCTURE software (Supplementary Table S2) confirmed the species assignments for all hatchlings tested using the multilocus plus mtDNA approach (Fig. 3a). The hybrid individuals analyzed with the 3RAD method were identified as backcrosses with loggerhead turtles (nests AB11 and AB17 in Fig. 3), confirming and further refining the hybrid class assignment obtained with multilocus data. The results for the different class assignments had 100% posterior probability support for all individuals according to the NewHybrids analysis (Fig. 3b).

### Analysis of morphology and reproductive success

**Figure 4:**
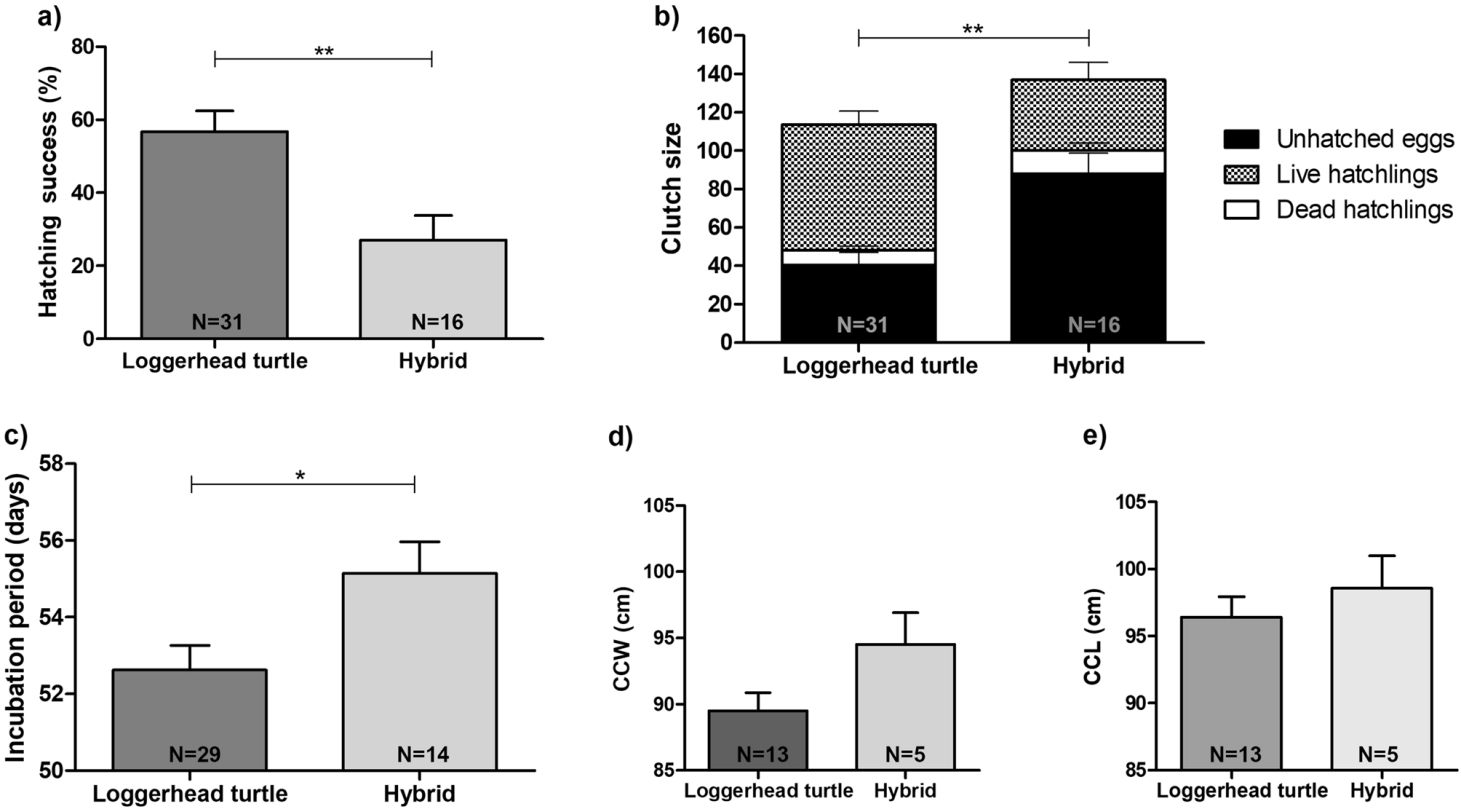
Graphical summary of morphological and reproductive parameters comparing loggerhead turtles and hybrids from the Abrolhos Archipelago. The hatching success (**a**), clutch size (**b**), incubation period (**c**), curved carapace width—CCW (**d**) and curved carapace length—CCL (**e**) are shown for both groups. Error bars show standard error. Statistical analysis was performed by nonparametric t test (Mann–Whitney U test). **P < 0.05; *P < 0.01.

Of the 20 different nesting females encountered on Abrolhos’ beaches over the four reproductive seasons between 2015 and 2019, 15 were identified as loggerheads and five as HxL hybrids, as described above. CCL and CCW values were estimated for 13 of the 15 loggerhead females and for all five female HxL hybrids. Average values of CCL were found to be 0.958 ± 0.05 and 0.986 ± 0.05, while average values of CCW were 0.891 ± 0.05 and 0.945 ± 0.05 for loggerhead and hybrid females, respectively. No statistically significant difference between these two female groups was found for CCW (Mann–Whitney U test p = 0.0676) and CCL measures (Mann–Whitney U test p = 0.2548), despite the fact that hybrids appear to be slightly larger and longer than pure loggerhead turtles (Fig. 4d,e).

**Figure 5:**
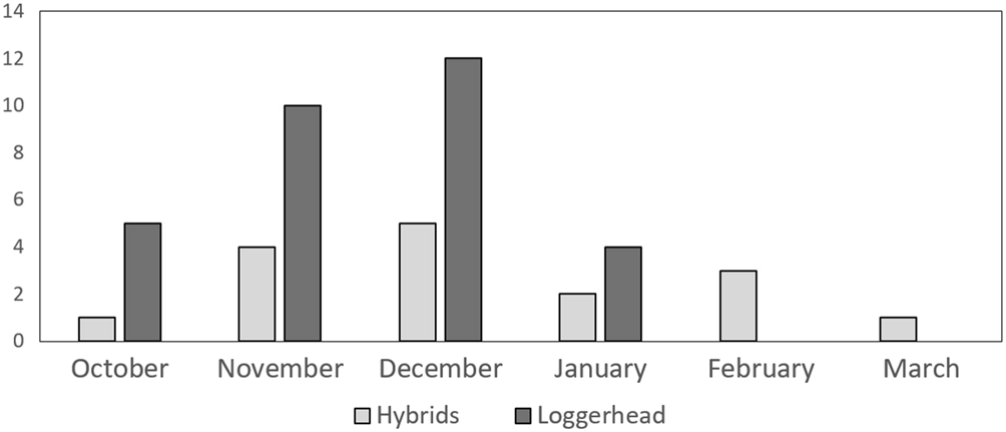
Temporal nesting distribution of hybrids (N = 16) and loggerhead turtles (N = 31) analyzed in this study.

We estimated the reproductive output for all 47 nests classified in this study (see Supplementary Figure S3 for a summary of tests performed per nest) and compared the reproductive output of the 31 loggerheads to the 16 hybrid nests. The hatching success was significantly greater (Mann–Whitney U test p < 0.01) for loggerheads (mean ± SD = 56.8 ± 31.3) compared to hybrids (27 ± 26.97) (Fig. 4a), showing that hybrids achieved lower reproductive success at the Abrolhos Archipelago. The clutch size was larger (Mann–Whitney U test p < 0.01) for hybrids (137 ± 20.06) than for loggerheads (113.7 ± 24.8) (Fig. 4b). The ratio of the total clutch size that corresponds to unhatched eggs was greater (Mann–Whitney U test p < 0.01) for hybrids (87.9 ± 43.4) in comparison to loggerheads (40.5 ± 36.1). Incubation period was significantly longer (Mann–Whitney U test p < 0.05) for hybrids (55 days ± 3.06) compared to loggerheads (52.6 days ± 3.5) (Fig. 4c). The temporal distribution of analyzed nests indicates that the reproductive season for loggerhead females finishes earlier than hybrids (Fig. 5).

## Discussion

### Abrolhos: a new hybrid area along the Brazilian coast

Hybridization between sea turtles has been previously reported at two nesting areas in Brazil with an extremely high frequency, reaching up to 42% of the hawksbill population of Bahia^11^. This work confirmed the presence of a new hybrid area in Brazil, with hybrid F1 HxL females nesting at the Abrolhos Archipelago and generating backcrossed progenies with both loggerhead and hawksbill males. Therefore, our results suggest that hybridization is a recent event in Abrolhos and support the previous study that estimated that the first hybridization event occurred at least one generation ago (i.e. 20 years)^13^.

Based on the mtDNA haplotype species assignment, our results indicate that the nesting hybrid female analyzed is the progeny of a female loggerhead and a male hawksbill. The same pattern was reported for the F1 hybrids from Bahia^10,13^, reinforcing the gender bias in crosses between loggerhead and hawksbill turtles.

Backcrossed hatchlings identified via a low throughput multilocus approach^13^ showed that F1 hybrids can reproduce with males of both parental species, indicating that the introgression process may be established in both directions, i.e., hybridization may potentially result in gene flow towards both parental species. It is well known that the Abrolhos Archipelago is a breeding area for loggerhead turtles, as well as a feeding, resting and cleaning symbiosis area for hawksbill turtles, which are attracted by the shallow fringing reefs^18–20,22^. Thus, opportunities for interspecific mating with both species are possible in Abrolhos—as our data indicate—assuming that nesting females are mating locally. Our results also suggest that hybrid females are not selective of parental species in their mate choice.

The reduced-representation genomic data obtained with a 3RAD approach^42^ confirmed the introgression level of some backcrossed hatchlings using 2,405 3RAD loci. This is the first time that backcrosses with loggerhead turtles are confirmed by genome-wide sequencing analysis. Previously, only backcrosses with hawksbills had been confirmed by multilocus analyses^10,13^, while confirmation of backcrosses with loggerheads had been suggested using only one nuclear marker^15^.

The first hybrid crossing that generated F1 female hybrids currently nesting in Abrolhos beaches may have originated between individuals from the Abrolhos population, if philopatric behavior is also expected for hybrids. Alternatively, these nesting hybrids could originally be from other neighboring nesting sites (such as Praia do Forte, Bahia). This is supported by the fact that nesting hybrids were recently reported (and confirmed by our investigation) in Abrolhos, and a hybrid female from Praia do Forte was shown to forage in habitats associated with coral reefs around Abrolhos^57^. This latter finding was reached by satellite tracking, which is a powerful tool for the understanding of sea turtle behavior and highly recommended to investigate the migratory, feeding and use of habitats of hybrids.

This is the first documentation of sea turtle hybrids nesting on islands. Indeed, the nesting of loggerhead turtles in the Abrolhos Archipelago, first reported in 1984^21^^,^ is also an atypical behavior, since their nesting beaches are typically wide and open beaches fronted by a flat approach from the sea and backed by low dunes^59^. The nesting monitoring of sea turtles in Abrolhos’ beaches has been performed since 2015, with the aim of clarifying important behavioral aspects such as the natal fidelity of females.

The high frequency of interspecific hybrids occurring in nesting sites in Brazil is an atypical event when compared to rookeries worldwide and is likely connected to the decreased chance of conspecific mating encounters due to the decline of sea turtle populations at the end of the twentieth century^10^. Considering the future projections of the deleterious effects of climate change and other anthropogenic pressures that may destabilize sea turtle populations^60^^,^ interspecific hybridization may increase in nesting areas used by different species. Thus, if hybridization is indeed a consequence of human-induced population decline and confirmed to be detrimental, as suggested by our results, targeted conservation management strategies, such as separating the rookeries of different species, will need to be developed. Additional populations should be analyzed in order to investigate the hybridization phenomenon between sea turtle species worldwide and its consequences for species conservation.

### Comparison between reproductive output of hybrid and loggerhead turtles

The hybrids presented larger clutch sizes relative to loggerhead turtles, which is usually correlated with body size, as the volume of eggs in a clutch is constrained by the volume within the hard shell of the female^14,61^. While we did not find significant differences in body size between hybrids and loggerheads, the previous analyses of Marcovaldi et al.^57^ and Soares et al.^14^ found that HxL hybrid turtles are larger than hawksbill and loggerhead turtles. Soares et al.^14^ hypothesized that either hybrids reach sexual maturity later or have faster growth rates than the parental species.

The average HS for loggerhead turtles at the Abrolhos Archipelago found in this study was 56.8%, which is lower than the loggerhead populations of Espírito Santo (79.9%), Bahia (73.1%) and Rio de Janeiro (76.74%)^24–26^. The landscape features of Abrolhos’ beaches help to explain lower rates of reproductive success in the archipelago^20^. Abrolhos’ beaches consist of narrow and short stretches of coral sand with extensive rock and grass coverage (Fig. 1). The beaches’ width ranges from 0 to 5 m, depending on the tide, with reefs obstructing offshore approach^62^. Studies have shown that large sand grain size and low vegetation cover positively influence sea turtle hatching success^63^^,^ while areas subject to tidal inundation are associated with lower success^64^. Addressing the influence of the landscape features and local conditions on hatchling viability could provide insights into the low reproductive rates observed in the archipelago.

The average HS for hybrids in Abrolhos was 27%, even lower than for loggerhead turtles. This demonstrated that the low overall reproductive success in Abrolhos is likely also related to hybridization, with a reproductive disadvantage for hybrids. This outcome is related to the greater number of unhatched eggs in the nests of F1 HxL hybrids in comparison to loggerhead turtles’ nests. However, the presence of embryos in the unhatched eggs was not checked. Future research should evaluate whether the low HS is associated with low egg fertility or embryonic mortality, two factors that can be responsible for the low success rate^65^.

Previous studies have shown reduction in the fitness of hybrids^66–68^, however, the underlying mechanisms have rarely been elucidated. Outbreeding depression usually occurs due to the disruption of local adaptation, the breakup of coadapted gene complexes, and/or the expression of hybrid incompatibilities^2^. The effect of outbreeding depression may be strongest in F1 hybrids, especially if the two hybridizing species have different karyotypes^28^. However, all sea turtle species have the same karyotype^69^ and therefore F1 hybrids must carry a haploid set of homologous chromosomes from each parental lineage. In such cases, the effect of outbreeding depression is often delayed until the F2 generation or later, when segregation and recombination begin to break apart coadapted genes from a single parental lineage^70^. In this work, the low hatching success was found for backcrossed hybrid nests, which suggests that outbreeding depression in sea turtles probably occurs starting from the second generation of hybrids. Fitness declines in F2 hybrids has been shown for other taxa, such as the tidepool copepod (*Tigriopus* *californicus*)^71^ and the largemouth bass (*Micropterus* *salmoides*)^72^.

Apart from carrying the same number of chromosomes, sea turtles are expected to have high levels of homology, largely conserved syntenic blocks and relatively low divergence in DNA sequences^73^. High conservation at the genomic level combined with deep phylogenetic splits make sea turtles a particularly interesting example of hybridization. Unhatched eggs from a backcrossed hybrid nest are likely to carry some type of genetic incompatibility across the genome, likely due to rearrangement of chromosomes and sets of incompatible genes present within or across chromosomes. Living hatchlings from the same nests are not necessarily viable for reproduction but have already been shown not to carry lethal combinations of genetic material. These individuals are valuable to the study of species-specific sea turtle genomic features, as they can provide insights into the potential for introgression between highly divergent sea turtle species and the areas of the genomes that are more prone to introgression. Such information is especially important for sea turtles, as their long generation times (20–40 years for loggerhead and hawksbill turtles^74,75^) would require vast lengths of time to determine population changes and track hybridization outcomes in nature.

Future impacts of hybridization in sea turtles will also depend on the establishment of the introgression process over generations, the onset of which was observed to be occurring in both directions of parental species. The continuous introduction of maladaptive alleles into threatened populations may lead to demographic and/or genetic swamping. As it will depend on the replacement rate, hybridization is a bigger threat to hawksbill turtles, which is the rarest species involved in hybridization events in Bahia. Previous studies analyzing the reproductive output of F1 HxL hybrid females compared to the parental species at Praia do Forte in Bahia, revealed that while emergence success was lowest for hybrids, hatchling production (the product of clutch size and emergence success) per clutch was similar among all groups^14^. In addition, the clutch size, incubation period, observed clutch frequency and observed breeding frequency were similar among all groups, suggesting no reproductive advantage or disadvantage of hybrids relative to their parental species. The proportion of viable hybrid hatchlings was also similar to ‘pure’ hatchlings^15^. These varying outcomes in different populations should be further investigated in order to elucidate the impact of hybrid fitness and inform conservation management.

Analyzing all 47 nests included in this study, we found that nesting events for loggerhead females finish earlier than for hybrids, since no loggerheads were found nesting in February and March, unlike hybrids (Fig. 5). Our temporal nesting distribution, although not systematically sampled, corroborates data from Soares et al.^14^ indicating that loggerhead females nest earlier than both hybrids and hawksbills, and that hybrids have a temporal nesting distribution that overlaps with both parental species. Hawksbill males arrive in the reproductive area of Bahia (Praia do Forte) around the loggerhead nesting peak (November and December), thus having the opportunity to mate with conspecific females as well as loggerhead females, which is the most abundant species on the Brazilian coast and in Abrolhos^10,23^. The timing of reproductive seasons of both species and the abundance of loggerheads along the Brazilian coast appear to favor interspecific crossings in Bahia^14^.

The different temporal distribution of nesting for loggerheads and hybrids likely influences the incubation period (IP), which was significantly longer for hybrids compared to loggerheads. IP is determined by the temperature at which a clutch develops, which varies during the reproductive season. Our study highlights the importance of combining ecological and genetic data to provide important insights into the effects of hybridization on sea turtle populations.

Considering that the estimated nesting frequency per female per season for loggerhead turtles is around four events^76,77^ and that the average number of nesting events per reproductive season in Abrolhos is 34^20^^,^ we were able to infer that the number of females nesting in Abrolhos per season is about nine. The low density of the nesting population may reflect a very low number of potential conspecific mates, favoring interspecific crossings. Despite its small population size, the Abrolhos population deserves thorough and continued study in order to understand the likely association between the hybridization process and reproductive success, which may impact sea turtle conservation.

The Abrolhos National Marine Park was created in 1983 with the aim to protect an area home to the richest marine biodiversity in Brazil. However, the conservation of this unique ecosystem is threatened by several factors, such as the illegal fishing activity, overfishing around the protected area, expanding tourism, dredging, invasive species, oil drilling and others^29,78^. More recently, a rupture of a tailings dam in Mariana (Minas Gerais, Brazil) caused a hazardous environmental catastrophe in the Doce River and estuary, reaching the nearby Abrolhos protection area and potentially contaminating the coral reef ecosystem^79^. These environmental stressors synergistically reduce the resilience of the environment and associated species. Here we present another important concern regarding sea turtle conservation at the Abrolhos Archipelago, where reduced hatching success is likely dependent on both environmental and genetic (interspecific outbreeding effects) influences.

## Supplementary information

Supplementary file1

Supplementary file2

Supplementary file3

## Data Availability

Original 3RAD data for this project have been deposited at the BioProject database under the ID PRJNA641778 with sample-specific accession numbers SAMN15366327–SAMN15366350. The datasets generated and analyzed during the current study are available in the supplementary material. All scripts used to analyze 3RAD data are available from GitHub (https://github.com/BeGenDiv/Arantes_et_al_2020).
