# Author Correction: Genomic evidence of recent hybridization between sea turtles at Abrolhos Archipelago and its association to low reproductive output

**DOI:** 10.1038/s41598-020-72367-y

**Published:** 2020-09-29

**Authors:** Larissa Souza Arantes, Lucas Cabral Lage Ferreira, Maximilian Driller, Fernando Pedro Marinho Repinaldo Filho, Camila Junqueira Mazzoni, Fabrício Rodrigues Santos

**Affiliations:** 1 Departamento de Genética, Ecologia e Evolução, Instituto de Ciências Biológicas, Universidade Federal de Minas Gerais (UFMG), Avenida Antônio Carlos, 6627, Belo Horizonte, MG 31270-010 Brazil; 2 Parque Nacional Marinho dos Abrolhos, ICMBio, Ministério do Meio Ambiente, Brasilia, Brazil; 3 Berlin Center for Genomics in Biodiversity Research (BeGenDiv), Königin-Luise-Straße 6-8, 14195 Berlin, Germany; 4 Evolutionary Genetics Department, Leibniz Institute for Zoo and Wildlife Research (IZW), Alfred-Kowalke-Straße 17, 10315 Berlin, Germany

Correction to: *Scientific Reports* https://doi.org/10.1038/s41598-020-69613-8, published online 30 July 2020

This Article contains a typographical error in the Acknowledgements section.

"This study was conducted with partial funds provided by CAPES and FAPEMIG Grant #APQ03625-2917 from Brazil, and by DAAD from Germany."

should read:

“This study was conducted with partial funds provided by CAPES and FAPEMIG Grant #APQ03625-17 from Brazil, and by DAAD from Germany."

