# Supplementary material for: Genomic evidence of recent hybridization between sea turtles at Abrolhos Archipelago and its association to low reproductive output
Source: Sci Rep. 2020 Jul 30;10:12847. doi: 10.1038/s41598-020-69613-8 (PMC7393485; doi:10.1038/s41598-020-69613-8)
Supplement: Supplementary file 3 — Supplementary file3 [file 41598_2020_69613_MOESM3_ESM.pdf]

Supplementary material

Genomic evidence of recent hybridization between sea turtles at Abrolhos Archipelago and its association to low reproductive output

Larissa Souza Arantes[1], Lucas Cabral Lage Ferreira[2], Maximilian Driller[3,4], Fernando Pedro Marinho Repinaldo Filho[2], Camila Junqueira Mazzoni[3,4*], Fabrício Rodrigues Santos[1*]

[1]Departamento de Genética, Ecologia e Evolução, Instituto de Ciências Biológicas, Universidade Federal de Minas Gerais (UFMG), Avenida Antônio Carlos, 6627, Belo Horizonte - MG, Brazil, 31270-010. Phone: +55 31 3409 2581.

[2]Parque Nacional Marinho dos Abrolhos, ICMBio, Ministério do Meio Ambiente, Brazil.

[3]Berlin Center for Genomics in Biodiversity Research (BeGenDiv), Königin-Luise-Straße 6-8, Berlin, Germany, D-14195. Phone: +49 (0)30 838 59961.

[4]Evolutionary Genetics Department, Leibniz Institute for Zoo and Wildlife Research (IZW), Alfred-Kowalke-Straße 17, Berlin, Germany, D-10315. Phone: +49 (0) 30 5168 0.

*Correspondence and requests for materials should be addressed to F.R.S (email: fsantos@icb.ufmg.br) and C.J.M (email: mazzoni@izw-berlin.de).

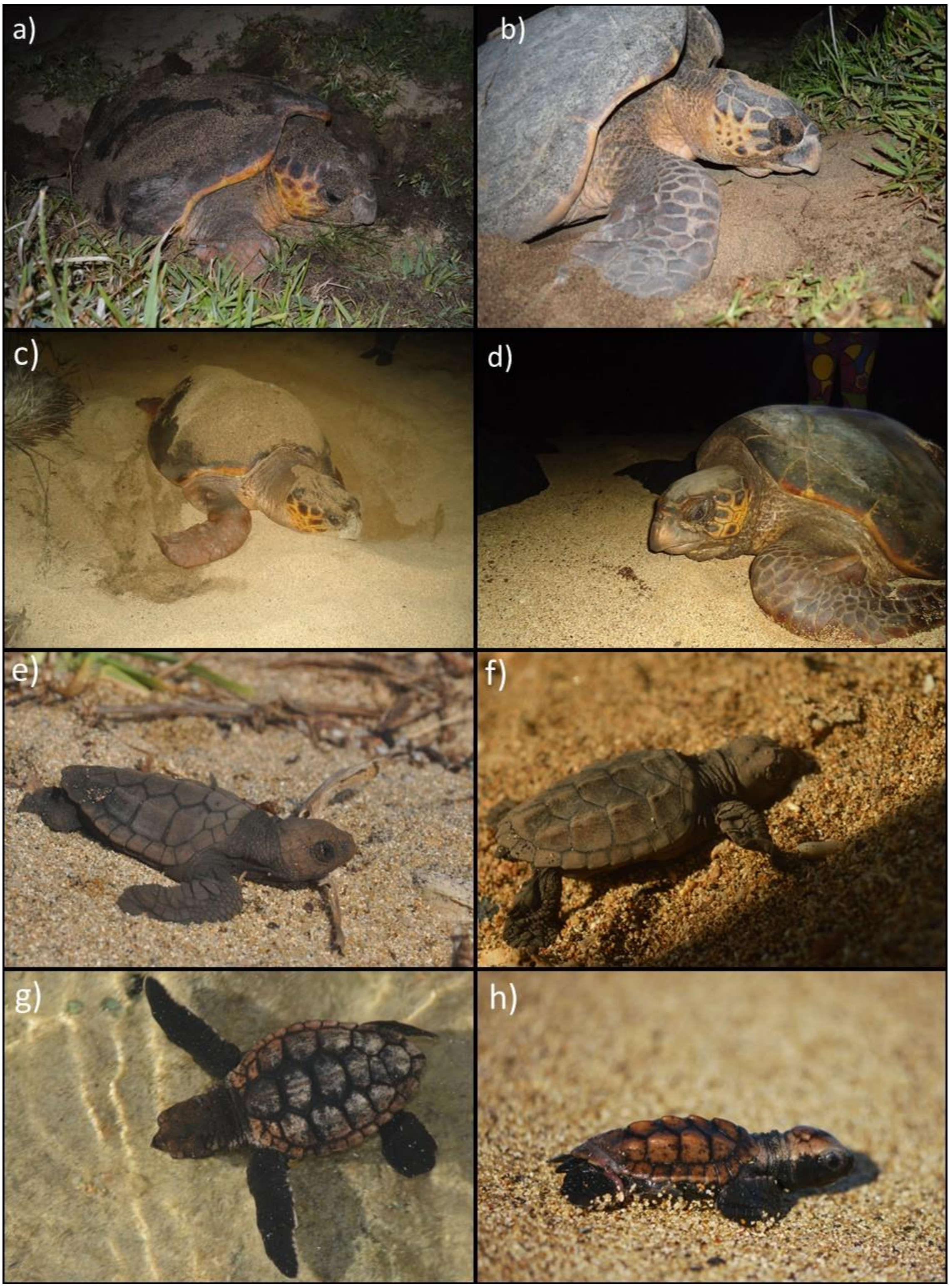

Figure S1 – Females and hatchlings of loggerhead turtles (left pictures – a, c, e, g) and loggerhead x hawksbill hybrids (right pictures – b, d, f, h). The pictures highlight the difference between "pure" and hybrid turtles in terms of the number of lateral scutes in the shell, the size of the head and the beak format.

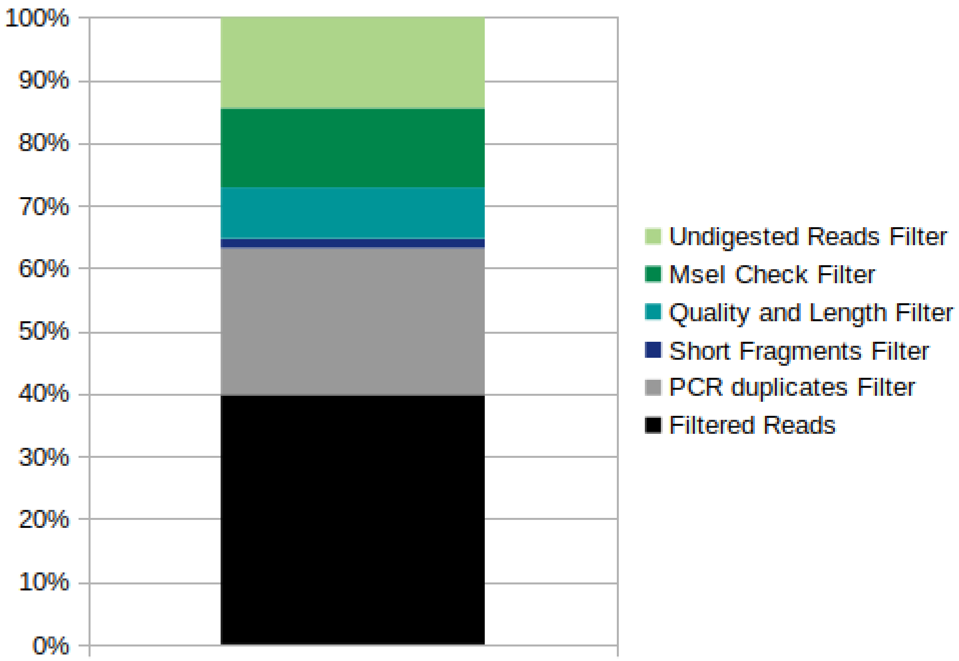

Figure S2. Percentage of reads removed from the 3RAD dataset in each filtering step. After the demultiplexing of samples, we filtered out the PCR replicates, short fragments (<240 bp), low quality reads (Q>30), undigested reads (read pairs containing internal complete restriction sites of *Mse*I, *Eco*RI and *Cvi*QI) and read pairs digested by *Cvi*QI (*Mse*I Check filter), leaving 39.7% of the reads (filtered reads) from the initial dataset.

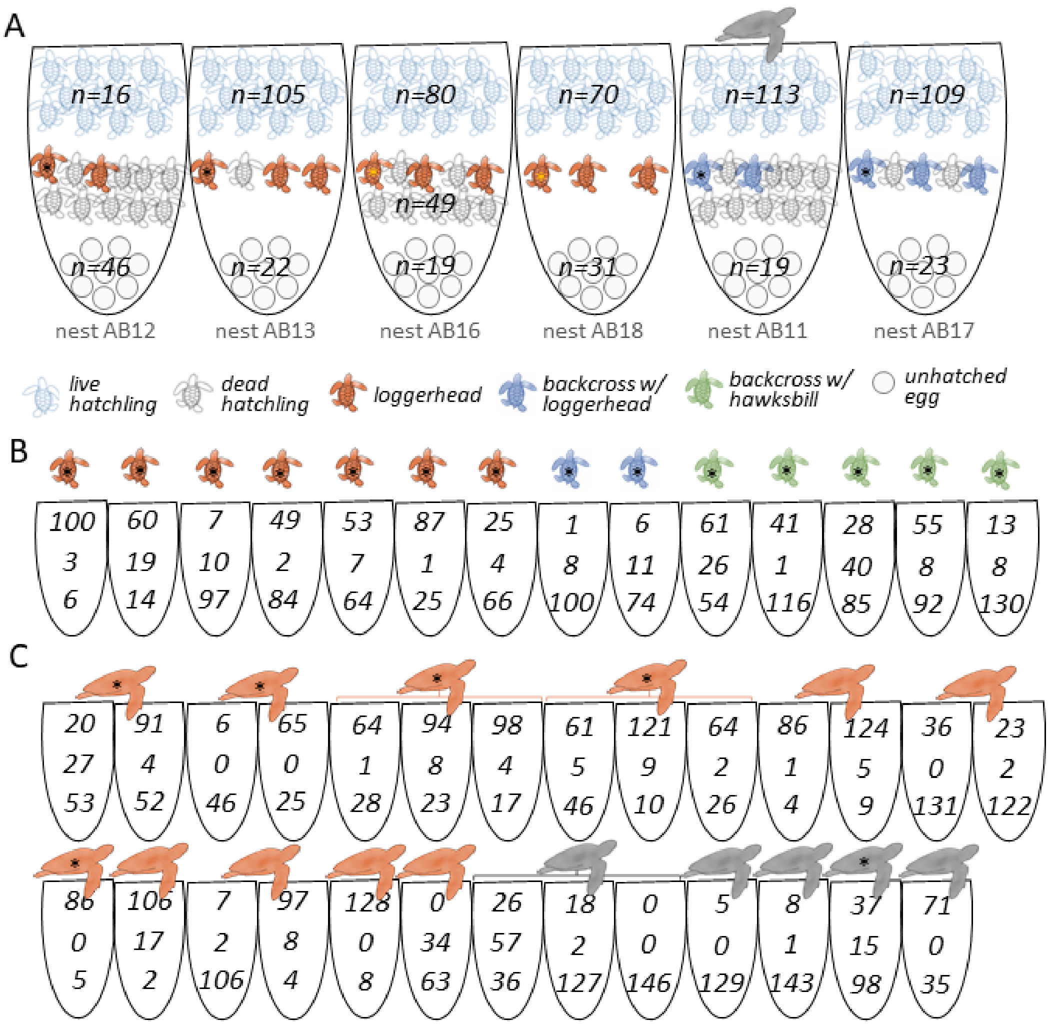

Figure S3. Representation of all nests analyzed in this study and the types of species and hybrid identification performed. Asterisks (*) represent results from multilocus analysis using Sanger sequencing from six nuclear markers. In (A) all 6 nests using both multilocus and 3RAD analysis are represented. Color-filled hatchlings have had their status confirmed by 3RAD (except for two individuals identified with a yellow asterisk, whose status was confirmed only by multilocus analysis). Whenever the number of hatchlings or eggs exceed the number of depicted figures, the total number (n) is indicated in the foreground. The female of nest AB11 (in gray) was encountered during spawning and morphologically identified as a hybrid. In (B), each representative dead hatchling tested from 14 nests and analyzed solely with the multilocus approach is colored according to the Hybrid Index classification (Figure 2). The number of live hatchlings, dead hatchlings and unhatched eggs are presented from top to bottom, as in (A). In (C), all remaining nests for which nesting females were morphologically identified as either loggerheads (in red) or F1 hybrids (in gray) are indicated. Multilocus analysis was always in agreement with 3RAD results as well as female morphological identification.
